# National Essential Medicines List and policy practice: A case study of China’s health care reform

**DOI:** 10.1186/1472-6963-12-401

**Published:** 2012-11-14

**Authors:** Xin Tian, Yaran Song, Xinping Zhang

**Affiliations:** 1 Tongji Medical College, Huazhong University of Science and Technology, 13 Hangkong Road, Wuhan, Hubei Province, 430030, P.R China; 2 Peking University School of Public Health, 38 Xueyuan Road, Beijing, 100191, P.R China

**Keywords:** Essential medicines list, Supplemented list, Policy

## Abstract

**Background:**

In 2009, China implemented the national essential medicines system by enacting the National Essential Medicines List 2009. According to the policy of this system, primary health care institutions can only stock and use essential medicines on the prescribed List. Meanwhile, each province can choose to make its own list of supplemented medicines. The goal of the study is to provide suggestions for emerging problems and identify future policy-making trends.

**Methods:**

In this study, we statistically analyzed the National Essential Medicines List 2009 and lists of supplemented medicines of all 29 provinces. We also examined the rationality of such medicines based on the DELPHI method and literature review, after which we studied the provincial supplements in relation to the national essential medicines system.

**Results:**

We demonstrated that the National Essential Medicines List 2009 provides a comprehensive coverage of diseases as well as reasonable varieties of drugs for their treatment. The average number of supplemented medicines in 29 provinces is 207, with each medicine included in 2.9 provincial lists on average. Only 2.6% supplemented medicines are included by more than half of the provinces (>15), indicating great regional variance. Among the 32 most frequently supplemented medicines, only 18 meet the selection principles, including two with strict usage restrictions.

**Conclusion:**

The structure and selection of the National Essential Medicines List 2009 are relatively reasonable. The main problems, however, include the excessive and non-scientific selection of medicines on the supplemented medicines list. The function of the provincial lists of supplemented medicines has not been achieved, which has influenced the effectiveness of the national essential medicines system in China.

## Background

Currently, 160 countries have formal essential medicines lists. More than 90% of the low- and mid-income countries have such lists, while the percentage for high-income countries has reached 60% [1,2]. The first edition of the Chinese Essential Medicines List, published in 1982, did not initially include Chinese traditional medicine [3]. In 1996, the List was revised to include both Western and Chinese traditional medicine. From then on, the List has been revised once every two years. In 2004, it included 2,033 essential medicines for the treatment of most common diseases, with 733 western drugs and 1260 Chinese traditional medicines. However, since the selection was excessive and only a few policies on essential medicines were in place, the lists did not play a significant role. Due to the fact that primary health care institutions were making profits from reselling medicines [4] and because of the widespread abuse of antibiotics and excessive use of parenteral [5-8], China published the National Essential Medicines List 2009 (the 7^th^ edition of the series), which marked the implementation of a national essential medicines system in the country. In China, the essential medicines system aims to (1) improve medicine availability, affordability and safety [9-11], and (2) cut the profit link between health institutions, doctors, and medicine (Table 1).

**Table 1:** **Number of medicines included in previous national essential medicines lists**[12]

**Edition**	**1982**	**1996**	**1998**	**2000**	**2002**	**2004**	**2009**
Western medicine	278	699	740	770	759	773	205
Chinese traditional medicine	0	1699	1333	1249	1242	1260	102
Total	278	2398	2073	2019	2001	2033	307

The essential medicines list is one of the core contents of the essential medicines system. The system requires that all government-run primary health care institutions be equipped with essential medicines merely, including those found on the National Essential Medicines List 2009 and on the respective lists of each province. This requirement restricts the medication made available in primary health care institutions, thus promote the rational use of medicines and reducing the opportunities for doctors to intentionally prescribe high-profit medicines. Such requirements, therefore, highlight the importance of scientifically selecting essential medicines because selection directly affects the treatment results.

This study mainly focuses on two key areas of the national medicine policy, namely, list making and policy implementation. Based on the National Essential Medicines List 2009 and the respective lists of supplemented medicines in the provinces, this study analyzes the list-making process and the policy optimization. In addition, we provide suggestions on how to better implement the essential medicines system in China and improve the use of such essential medicines. This study also provides references on policies and approaches for other countries that can guide the creation of essential medicines list and the implementation of the essential medicines system.

## Methods

### Case study

Since 2009, China has reconstructed the National Essential Medicines List, issued supporting policies, granted manufacturing licenses, and enacted requirements to guide the use of such essential medicines. The innovative measures related to the national essential medicines practice in China are worth analyzing and discussing. The information generated can then provide a reference for other countries in similar situations.

### Data collection and analysis

This study analyzed the National Essential Medicines List 2009 and all other lists from 29 provinces. The data used in the manuscript were gathered from the Internet. Using statistical calculation and canonical analysis, we evaluated the rationality of the structure and category of the selected medicines at the theoretical level.

The statistical analysis included three indicators. (1) The first is the number of medicines, which included two parts, i.e., the average number of the supplemented medicine by provinces and the number of each category of medicine on the National Essential Medicines List and on the lists of supplemented medicines based on their effects. (2) The second indicator is the frequency of the same medicine appearing on different supplemented lists, which indicates the frequency of supplementation of one category of medicine. For example, if one category of medicine is supplemented by one province, its frequency of supplementation is “1.” If it is included by two provinces, its frequency of supplementation is “2.” (3) The third indicator is the co-selection rate, which is the quotient of the total number of supplemented medicines from 29 provinces and the number of unique medicines on such lists. This means how many provinces on average every supplemented medicine was selected by.

This study focuses on the rationality of the structure and category of selected medicines from the National Essential Medicines List as well as the supplemented medicines using the DELPHI method and literature review. Delphi method provides a credible and scientific way to answer certain questions. Experts do not know each other and they answer questions in back-to-back style to ensure the impartiality of the results. The contents of the questionnaire are designed to evaluate each drug on the list. The experts included five long-time pharmacy experts and five senior general practitioners. Two rounds of questionnaire surveys were conducted. Each expert is expected to give their opinion as to “whether (or not) the drug meets the supplementation criteria” and “supporting evidence.” As for the rationality of the selected medicines category, the analysis was made using 205 categories of Western medicine and 32 most frequently supplemented medicines by 29 provinces (frequency ≥ 16). This analysis was based on four indicators: (1) primary clinical necessity, (2) clinical effectiveness, (3) adverse effect, and (4) competitive price. The rationality and necessity of each category were evaluated according to the results of all four indicators based on the survey from the experts. As for the structure of the lists, a survey was conducted on whether or not the National Essential Medicines List and the supplemented medicines had comprehensive coverage and a reasonable structure based on the number of medicines in each category. The results from the first round of surveys were listed as references for experts for the second round. After two rounds, all experts reached a consensus on each category.

Meanwhile, face-to-face interviews with five medicine policy-making experts were conducted. We used a semi-construction interview on the process of selecting the supplemented medicines in every province, the policy evidence of the current essential medicines system and the emerging problems and solutions.

Based on results of the analysis, we assessed the practicality and logicality of the policies and measures of the essential medicines system of China. We also provided analyses and suggestions on the emerging problems in the future.

## Results

### The National Essential Medicines List 2009

#### Rationality of the list structure

The National Essential Medicines List 2009 includes 307 medicines comprising 205 chemical medicines covering 24 categories of diseases and 102 Chinese traditional medicine covering six categories of clinical indications. A large proportion of the medicines are used to treat diseases with high prevalence in China, such as diseases of the respiratory, cardiovascular, and digestive systems. Some chronic diseases that have rapidly spread recently, such as hypertension and diabetes mellitus, are also covered, along with common illnesses such as cold. The comparatively comprehensive structure and categories of the essential medicines list meet the basic medical needs in China.

However, we found a few problems. Several categories of medicines are missing in the structure. First, although there are medicines to treat certain symptoms of common diseases (e.g., cold, fever, abdominal pain, and diarrhea), there are not enough common compound preparations to provide comprehensive relief. Second, medicines for chronic diseases (e.g., cardiovascular and nervous system diseases) are also lacking. Third, some primary health care institutions provide specialty services that require medicines for dermatology, orthopedics, gynecology, and so on. There are only 5 dermatology drugs on the National Essential Drugs List, a mere 1.63% of the entire list consisting of 307 varieties. The provinces added as many as 47 such products to their lists, representing 2.96% of the total. There are only 8 orthopedic medicines on the list, accounting for 2.6% of the total, while the provinces added up to 70 such products, accounting for 4.4% of supplemented total. These figures indicate that the categories in the National Essential Drugs List cannot meet the specific service needs of primary health care institutions. The same is true for gynecological medicine, three of which appear on the National Essential Drugs List. Finally, medicines for children as well as information on the appropriate dosages are also insufficient. Few options exist in China in terms of pediatric drugs and dosage forms, and the list of essential medicines for children has yet to be introduced [13-15]. In fact, there are only 60 medicines and dosage forms available for pediatric use among more than 3000 varieties of pharmaceutical preparations in China, representing less than 2% of the total [16].

#### Rationality of the selected categories

In this aspect, experts questioned the safety and rationality of pethidine and colchicines. The metabolite of pethidine is norpethidine, which has a long half-life in vivo, and can lead to mental disorders, tremors, or convulsions because of its toxicity. Thus, its cost effectiveness is lower than other medicines in the same category, such as morphine and diazepam. Colchicine can cause clinical adverse events, including stomachache, diarrhea, vomiting, anorexia and other gastrointestinal reactions, with an incidence rate of 80%. The rest of the medicines can meet the requirements of “safety, necessity, effectiveness, and competitive price.”

### Lists of supplemented medicines of 29 provinces

#### Rationality of the list structure

The supplemented lists from 29 provinces include 2062 medicines that comprise 783 chemical medicines and 805 Chinese traditional medicines, with an average of 207 per province. The lists also include 352 traditional Tibetan medicines in Tibet and Qinghai Province, and 122 Mongolian medicines in Inner Mongolia.

The supplemented medicines cover all the categories found on the National Essential Medicines List as well as a new category for antineoplastic medicines. Medicines for the digestive system and for the cardiovascular system, as well as those for antimicrobial, antipyretic, analgesic anti-inflammatory, and anti-rheumatic purposes account for 42.7% of the supplemented chemical medicines in the List. A similar trend is also present in Chinese traditional medicines. The abovementioned structure makes up for the deficiency of the National Essential Medicines List (Table 2).

**Table 2:** **Distribution of medicines in the national essential medicines list and in the supplemented medicines lists** (**all the supplemented medicines by 29 provinces**)

**No.**	**Category**	**No. of national essential medicines**	**No. of supplemented medicines**
C1	Antimicrobial medicine	33	95
C2	Antiparasitic medicine	7	7
C3	Narcotic	4	15
C4	Antipyretic and analgesic anti-inflammatory and anti-rheumatic medicine	9	77
C5	Medicine for nervous system	14	48
C6	Medicine for mental disorder	6	21
C7	Medicine for cardiovascular system	29	66
C8	Medicine for respiratory system	7	34
C9	Medicine for digestive system	17	96
C10	Medicine for urinary system	5	13
C11	Medicine for hematologic system	10	45
C12	Hormone and medicine affecting the endocrine system	15	50
C13	Anti-allergy medicine	4	5
C14	Medicine for immune system	2	14
C15	Vitamin, mineral substance medicine	7	34
C16	Medicine for humoral regulation	8	10
C17	Antidote	5	16
C18	Biological product	4	8
C19	Diagnostic agent	2	10
C20	Medicine for dermatology	5	47
C21	Medicine for ophthalmology	5	24
C22	Medicine for otorhinolaryngology	3	9
C23	Medicine for gynecology	3	9
C24	Medicine for birth control	1	5
C25	Antineoplastic medicine	0	25
T1	Internal medicine (diaphoresis, clearing heat, and relieving cough)	24	212
T2	Internal medicine (cardiovascular system)	16	153
T3	Internal medicine (digestive system)	20	121
T4	Internal medicine (other)	13	94
T5	Surgery	7	32
T6	Gynecology	8	62
T7	Ophthalmology	2	9
T8	Otorhinolaryngology	4	38
T9	Orthopedics and Traumatology	8	70
T10	Dermatology	0	14
	Total	307	1588

Note: A kind of medicine for multi-diseases is counted only once.

C = Chemical medicines, T = Chinese traditional medicine.

#### Rationality of the selected categories

On average, each medicine is included by only 2.9 provincial lists, indicating a rather low co-selection rate. Based on the analysis, among the 2064 supplemented medicines, only 41 are listed by more than half of the provinces (> 15). These 41 supplemented medicines include 32 chemical medicines and 9 Chinese traditional medicines that account for just 2.6% of the total. Of the 783 chemical medicines, 319 appear on only one supplemented list, whereas 588 appear on less than five lists, accounting for 75% of the total. Of the 805 Chinese traditional medicines, 428 appear on only one supplemented list, whereas 712 appear on less than five lists, accounting for 91% of the total. The rates clearly show that the categories of medicine in each province differ greatly and follow no rules.

In terms of the rationality of the medicine selection, we analyzed 32 of the most frequently supplemented medicines based on the principle of “safety, necessity, effectiveness, adverse effect, and competitive price” using the DELPHI method and literature review. Only 18 supplemented medicines meet the selection criteria, including 2 with strict usage restrictions (Table 3).

**Table 3:** Analysis of the frequently supplemented chemical medicines

**No.**	**Name**	**Frequency**	**Suggestion**	**Evidence**
1	Colloidal Bismuth Pectin	16	Supplement	Meets the selection principles
2	Gliclazide	16	Supplement	Meets the selection principles
3	Chymotrypsin	17	Supplement	Meets the selection principles
4	Amlodipine	17	Supplement	Meets the selection principles
5	Cefaclor	17	Supplement	Meets the selection principles
6	Calamine	17	Supplement	Meets the selection principles
7	Morphine	18	Supplement	Meets the selection principles
8	Loratadine	20	Supplement	Meets the selection principles
9	Triamcinolone acetonide	20	Supplement	Meets the selection principles
10	Acarbose	20	Supplement	Meets the selection principles
11	Vitamin B complex	20	Supplement	Meets the selection principles
12	Cefotaxime	20	Supplement	Meets the selection principles
13	Tinidazole	22	Supplement	Meets the selection principles
14	Etamsylate	22	Supplement	Meets the selection principles
15	Flunarizine	24	Supplement	Meets the selection principles
16	Cefradine	24	Supplement	Meets the selection principles
17	Clotrimazole	24	Supplement external preparation only	Have significant side effects by oral administration, while effective as external anti-fungal medicine
18	Fluocinolone acetonide	17	Supplement external preparation only	Have significant side effects by oral administration, while effective as external anti-inflammatory medicine
19	Somidon	29	Cannot supplement	Have significant side effects
20	Cimitidine	24	Cannot supplement	Have significant side effects
21	Diethylstilbestrol	18	Cannot supplement	Have significant side effects
22	Ofloxacin	16	Not supplement	Levofloxacin, the levorotatory form of ofloxaxin, is included in the essential medicines list; it is more effective and less expensive.
23	Ambroxol	16	Not supplement	Oral dosage of ambroxol is included in the essential medicines list. The injection only applies for emergencies, and it is more expensive.
24	Coenzyme A	16	Not supplement	Mainly used for leucopenia and functional hypothermia. Disputes exist on its effect.
25	Vitamin C	16	Not supplement	Injection is included in the essential medicines list. The oral dosage is OTC medicine; therefore, it is not suggested to be supplemented.
26	Piracetam	17	Not supplement	No sufficient clinical- or evidence-based medical evidence to prove its effectiveness
27	Troxerutin	18	Not supplement	No sufficient clinical- or evidence-based medical evidence to prove its effectiveness
28	Lincomycin	19	Not supplement	Similar effect with clindamycin in the essential medicines list, and no clinical advantage
29	Oryzanol	21	Not supplement	No sufficient clinical- or evidence-based medical evidence to prove its effectiveness
30	Furazolidone	22	Not supplement	No sufficient clinical- or evidence-based medical evidence to prove its effectiveness
31	Roxithromycin	22	Not supplement	Same antibacterial spectrum with erythromycin in the essential medicines list; more expensive
32	Inosine	24	Not supplement	No sufficient clinical- or evidence-based medical evidence to prove its effectiveness

Several publications have reported adverse events of certain supplemented medicines. Some drugs with serious adverse events are eliminated products from other countries. These unsafe medicines can harm the health of an individual when listed as essential medicines.

## Discussion

### Main problems of the National Essential Medicines List and the lists of supplemented medicines implemented in each province

Overall, the National Essential Medicines List is scientific and reasonable. Despite a few problems, it has played a positive role in guaranteeing the availability of essential medicines, regulating medical services, and promoting rational medication. One study from Peking University showed that the available rate of a commonly used drug does not exceed 40% before the system is implemented; however, after the initial implementation at 20 provinces, they found that the availability rate increased to about 60% after conducting a survey between February-March 2010 [17]. Statistical data from State Council Medical Reform Office indicated that the average price at primary health care institutions dropped by 16.9%. Expense of single prescription and the prescription rate of antibiotics decreased by 9.0% and 1.38% after the reform in township hospitals, respectively.

The provincial lists of supplemented medicines have problems in terms of number, structure, and category of medicine. First, the selection is excessive and does not follow strict selection principles. The average number of supplemented medicines in each province equals to 67% of the entire List; such excessiveness weakens the function of the National Essential Medicines List. Second, some categories of supplemented medicines are not selected scientifically; thus, these lists of supplemented medicines show characteristics of “excessiveness, dispersion, and disorder.” Many clinically non-preferred medicines with serious side effects are introduced into primary care health institutions due to the unscientific selection approach. For example, somiton is the only medicine listed by all 29 provinces. The aminopyrine found in somiton causes hypoleukocytosis, which resulted in 1,981 deaths in the U.S. from 1931 to 1934, prompting the U.S. government to eliminate it from the legal medicines list in 1938. Another ingredient of somiton is phenacetin, which has been proven to be one of the causes of renal failure and is already restricted in many countries [18,19]. Meanwhile, cimitidine is listed by 24 provinces and ranks second in supplementing frequency; this has complicated pharmacological effects and multiple side effects. It can also accentuate the pharmacological activity or toxicity of other medicines through drug-drug interactions. Therefore, cimitidine is usually replaced with ranitidine [20,21]. Diethylstilbestrol is listed by 18 provinces as a supplemented medicine for estrogen supplement. However, it can cause serious adverse events, including carcinoma of the vagina in young girls. Such major adverse events have made diethylstilbestrol an uncommon choice in large hospitals both at home and abroad [22].

Form the literature and interview date, almost all of the 29 provinces based their selections on various experts’ opinions in producing their respective lists of supplemented medicines, and a majority of experts are from the prime health care institutions. In comparison, the selection of the WHO Lists of Essential Medicines is mainly based on evidence-based medicine and pharmaceutical economics – opinions of experts are only considered as references. Currently, only a few provinces (e.g., Sichuan) apply the method of evidence-based medicine selection and consider the disease status in common patients.

### The function of the provincial lists of supplemented medicines and their actual effects

Under the regulation of the essential medicines policy in China, and due to the concern that the National Essential Medicines List may not meet all the needs of primary health care institutions, the policy now allows province to add to the List those drugs that are associated with current use at the first stage of the reform. Provincial supplemented medicines should be limited to local use for special diseases, and their usage must coincide with the functions and positions of primary health care institutions. Aims of the provincial supplementing policy are include meeting the rational needs of prime health care institutions, and cutting the financial ties between doctors and drug manufacturers, gradually changing the medication practices of doctors, promoting the rational use of medicine, that same to essential medicines system.

However, the goal of this policy has not yet been achieved. On one hand, even with regulation and revisions of the principles and methods of supplementation, the provincial supplemented lists are still unreasonable and random. This is because the provinces have been creating their own lists based on the existing medication practices of their respective health care institutions and doctors. Moreover, most provinces do not have a guideline on the proportion of the supplemented medicines. These greatly have reduced the effectiveness of the essential medicines system in promoting the rational use of medicine and changing the medication practices of doctors. The fact that doctors still use the same medicines makes it impossible to cut the financial ties between doctors and drug manufacturers. In turn, this makes it more difficult to meet the goal of the essential medicines system.

On the other hand, even after the provinces added essential drugs, the primary health care sector still cannot meet the actual needs of doctors and patients [23,24]. The total number of medicines available is still high despite the fact that the List lacks some commonly used drugs that vary in diverse regions, at various institutions, and with some doctors.

Although provincial lists of supplemented medicines are unable to reach their original targets, revising the National Essential Medicines List should be based on the supplementing experience of each province; furthermore, frequently supplemented medicines with clear pharmacological benefits should be added. After revising, the List should meet the most basic medication needs of primary health care institutions in terms of both the category and the number of the medicines.

### Recommendations on usage policy of essential medicines

The usage policy of essential medicines is one of the key measures of the essential medicine system in China. The provincial supplementing policy is based on usage policy, which is related to the selection and function of National Essential Medicine List and the provincial lists of supplemented medicines. Thus, it is necessary to discuss the current usage policy, despite the fact that it does not directly follow from the data in research results. The usage policy of essential medicines should also be adjusted. Doctors in primary health care institutions should not be limited to prescribing only the essential medicines and should enjoy some autonomous rights within a certain range.

A country that controls the prescription practices of doctors with an essential medicines list covering a few medicine categories, in order to reduce medicine cost and promote rational medication is rare. This rarity is due to the specificity of the medical industry and the individual variations within it. The existence of a reasonable enough list that suggests the best treatment therapy for every patient is impossible because of the limitation of categories and the number of essential medicines. Apart from the fact that medication needs in different areas are not the same, the limitation resulting from the essential medicines list is in conflict with the current medical insurance system in China, given that the list just covers less than 1/7 of the Basic Medical Insurance Directory. Limiting the medication of all health care institutions and doctors to the same list of medicines can be a short-term measure, but such a policy is inappropriate for the situation in China and incompatible with the law of medicine as a whole. This practice can also cause conflicts between the prescription practice of doctors and the medical needs of patients, resulting in great decrease of doctors’ enthusiasm and patients’ satisfaction. In fact, these events have happened in some areas in China.

Analyzing the issue from another angle, the aim of designing the usage policy is improving rational use and cutting the profit link between health institutions, doctors, and medicine. However, there are many factors in China that cause irrational drug use. These factors mainly include the following: (1) medical personnel do not have the required level of knowledge and capability and may even have poor prescription habits; (2) financial interests among pharmaceutical firms, hospitals and doctors (i.e., hospitals profit by reselling drugs); (3) patients demand inappropriate use of medicines due to their limited medical knowledge and long-term doctor-induced habits. When many patients do not obtain the desirable outcomes, they often demand the use of antibiotics and parenteral. Current usage policy may not improve rational use.

As for the issue of cutting the financial ties between doctors and drug manufacturers and then solving the problem of the health care system, simply depending on the essential medicines system is not enough–more comprehensive efforts are needed. First, the management mechanism, compensation model, and income distribution mechanism in health institutions should be transformed. Second, relevant departments should strengthen the supervision on the diagnosis and treatment behavior of health institutions and health workers. Third, the production and circulation of medicines should be regulated, including the restriction of medicine approval, control of medicine prices, and circulation cost. Finally, the mode of medical insurance payment should be turned from post payment to the imprest system. The current usage policy cannot obtain its goal and would only be able to do so with the above combined efforts.

## Conclusion

The National Essential Medicines List 2009 is fairly reasonable in terms of the number and structure of medicine. However, the lists of supplemented medicines by each province are excessive and are not so rational. These lists have been created selected based on the existing practices of local health institutions and doctors, and such selection process prevents he essential medicines system from promoting the rational use of medicine and ensuring the availability and affordability of essential medicines and drug safety. China will revise the National Essential Medicines List in 2012. The new selection and correlative policy should learn from the provincial experience.

## Competing interests

The authors declare that they have no conflicting interests, and there are no ethics problems met with this study.

## Authors' contributions

XT designed the study, provided input into the data analysis and interpretation, and wrote the first draft of the manuscript. YRS contributed to the study design, handled the data collection and analysis, and helped write the first draft. XPZ led and supervised the study, contributed to the study design, and assisted in the writing of the first draft. All authors read and approved the final manuscript.

## Pre-publication history

The pre-publication history for this paper can be accessed here:

http://www.biomedcentral.com/1472-6963/12/401/prepub
